# Yersiniabactin Reduces the Respiratory Oxidative Stress Response of Innate Immune Cells

**DOI:** 10.1371/journal.pone.0008240

**Published:** 2009-12-29

**Authors:** Armand Paauw, Maurine A. Leverstein-van Hall, Kok P. M. van Kessel, Jan Verhoef, Ad C. Fluit

**Affiliations:** Department of Medical Microbiology, University Medical Centre Utrecht, Utrecht, The Netherlands; University of Toronto, Canada

## Abstract

*Enterobacteriaceae* that contain the High Pathogenicity Island (HPI), which encodes the siderophore yersiniabactin, display increased virulence. This increased virulence may be explained by the increased iron scavenging of the bacteria, which would both enhance bacterial growth and limit the availability of iron to cells of the innate immune system, which require iron to catalyze the Haber-Weiss reaction that produces hydroxyl radicals. In this study, we show that yersiniabactin increases bacterial growth when iron-saturated lactoferrin is the main iron source. This suggests that yersiniabactin provides bacteria with additional iron from saturated lactoferrin during infection. Furthermore, the production of ROS by polymorphonuclear leukocytes, monocytes, and a mouse macrophage cell line is blocked by yersiniabactin, as yersiniabactin reduces iron availability to the cells. Importantly, iron functions as a catalyst during the Haber-Weiss reaction, which generates hydroxyl radicals. While the physiologic role of the Haber-Weiss reaction in the production of hydroxyl radicals has been controversial, the siderophores yersiniabactin, aerobactin, and deferoxamine and the iron-chelator deferiprone also reduce ROS production in activated innate immune cells. This suggests that this reaction takes place under physiological conditions. Of the tested iron chelators, yersiniabactin was the most effective in reducing the ROS production in the tested innate immune cells. The likely decreased bacterial killing by innate immune cells resulting from the reduced production of hydroxyl radicals may explain why the HPI-containing *Enterobacteriaceae* are more virulent. This model centered on the reduced killing capacity of innate immune cells, which is indirectly caused by yersiniabactin, is in agreement with the observation that the highly pathogenic group of *Yersinia* is more lethal than the weakly pathogenic and the non-pathogenic group.

## Introduction

*Yersinia pestis* is regarded not only as a potential bioterrorism threat, but also as a re-emerging pathogen [1]. To combat this potential threat, understanding the pathogenesis of *Yersinia* species is crucial. *Yersinia* spp. can be divided into a high-pathogenicity group (*Y. pestis*, *Yersinia pseudotuberculosis*, and *Yersinia enterocolitica* biogroup 1B), a low-pathogenicity group (*Y. enterocolitica* biogroups 2 to 5), and a non-pathogenic group (*Y. enterocolitica* biogroup 1A) on the basis of the lethal infectious dose in a mouse model [2], [3]. Of these groups, only the high-pathogenicity group contains the High Pathogenicity Island (HPI) [2], [3]. This HPI is generally adjacent to the type III secretion system secreted *Yersinia* outer proteins (Yops), an essential factor for the full virulence of *Y. pestis* in mammals [4]–[6]. The HPI was first described in *Yersinia* spp., but it has also been described in other *Enterobacteriaceae* [7]–[12]. Similar to *Yersinia* spp., *Escherichia coli* and *Klebsiella pneumoniae* isolates that contain the HPI are more virulent than isolates that lack the island [4], [12]–[16]. Furthermore, the HPI was highly associated with an *Enterobacter hormaechei* outbreak strain (EHOS) that caused a nation-wide outbreak in The Netherlands [17]–[19]. The HPI encodes for an effective siderophore, yersiniabactin. Yersiniabactin is synthesized by a complex assembly line in which YbtS, YbtE, HMWP1, HMWP2, and YbtU are essential proteins [20], [21]. HMWP1 and HMWP2 (High Molecular Weight Protein 1 and 2) are 350 and 230 kDa proteins that are encoded by *irp1* and *irp2*, respectively [22], [23]. In addition to these factors, other genes are involved in the regulation and transport of yersiniabactin, such as the *fyuA* gene, which encodes the yersiniabactin receptor.

Yersiniabactin facilitates iron uptake, which is essential for bacterial growth. In an environment with very limited free iron, such as the human body, where most iron is bound to proteins, bacterial expression of the HPI genes has been shown to be elevated [24], [25].

Iron plays an important role in the innate immune system. With the exception of a very limited amount of free iron, in mammals most iron is found incorporated into complex molecules, such as hemin, or bound to transferrin and lactoferrin. During infection, polymorphonuclear leukocytes (PMNs) upregulate lactoferrin production, which reduces the availability of free iron for bacteria and increases the availability of lactoferrin-containing iron for PMNs [26]. PMNs release the lactoferrin-containing iron into their phagocytic vacuoles where iron(II) can function as a catalyst to produce hydroxyl radicals (•OH) from hydrogen peroxide (H_2_O_2_) and superoxide anions (O^–^_2_) via the Haber-Weiss reaction [27]–[29]. Hydroxyl radicals are highly reactive and one of the most potent oxidants of the reactive oxygen species (ROS) produced by phagocytic cells [27]–[29]. However, conclusive evidence that the Haber-Weiss takes place under physiological conditions and that hydroxyl radicals are produced is lacking [30]. The endogenous production of hydroxyl radicals by PMNs is in doubt because the specificity of the experiments has been questioned, and it has been argued that exogenous iron in the buffers used during *in vitro* experiments could catalyze the production of hydroxyl radicals rather than endogenous production in PMNs [31]. Whether monocytes or monocyte-derived macrophages are also dependent upon iron provided by lactoferrin for the production of hydroxyl radicals is unclear, but they are able to produce hydroxyl radicals when activated, where exogenous Fe(III) can serve as a catalyst [32], [33].

The aim of our study was to investigate the possible role of yersiniabactin in virulence.

Growth experiments with a wild-type, yersiniabactin-producing strain (E4) and a yersiniabactin-negative *irp2* mutant derived from E4 were performed to determine which human-derived iron source was beneficial for the growth of yersiniabactin-producing bacteria. Eighty percent iron-saturated lactoferrin supported the growth of the wild-type E4, but not the *irp2* mutant. Because the increased virulence of yersiniabactin-producing bacteria could not solely be explained by the increased iron uptake and growth, we hypothesized that yersiniabactin may affect the innate immune system [10]. Previous work has demonstrated a dual role for another siderophore, deferoxamine: deferoxamine provided iron to *Y. enterocolitica* and modulated the innate immune system [34], [35]. Furthermore, we hypothesized that PMNs use iron for the production of hydroxyl radicals [28]. Therefore, we speculated that yersiniabactin may also play a role in reducing the innate immune response. This hypothesis was investigated by testing the effect of yersiniabactin and other iron chelators on the ROS production of activated innate immune cells. Of the tested iron chelators, ROS production by innate immune cells was most inhibited by yersiniabactin.

## Results

### Dual Role for Yersiniabactin

**Figure 1:**
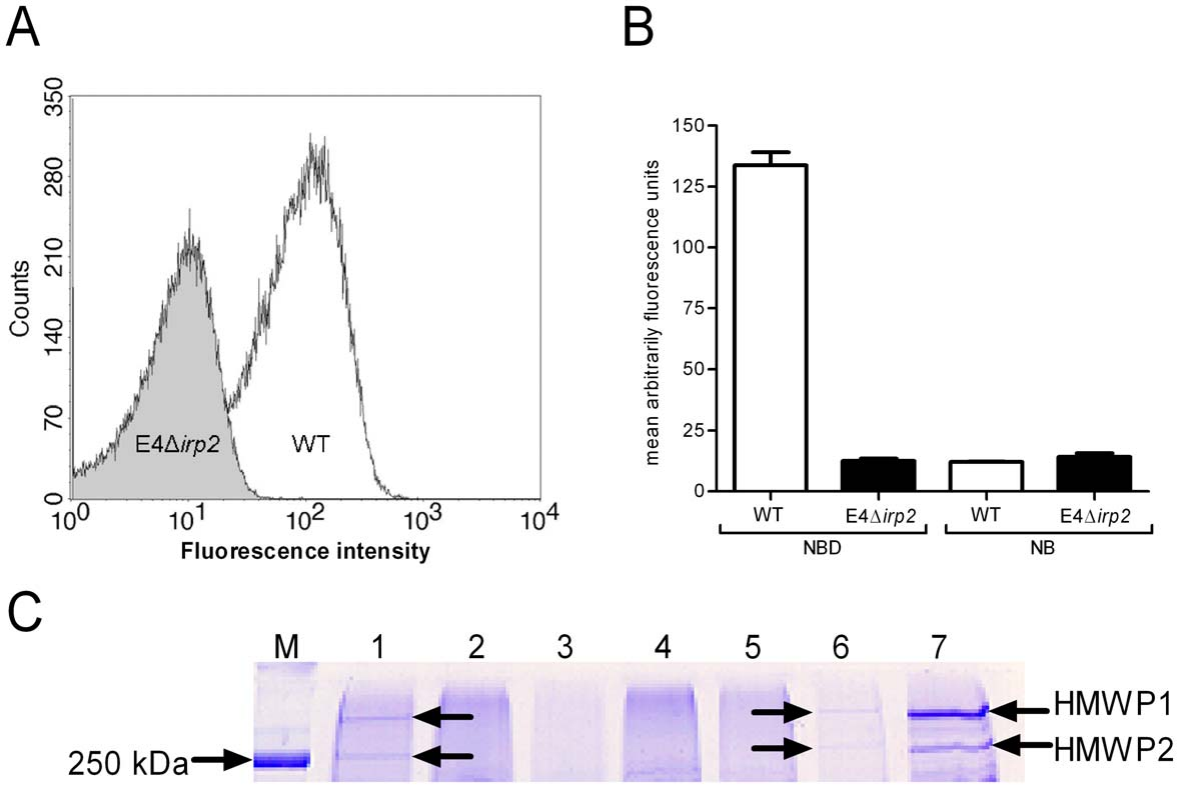
In the *irp2* knockout strain the production of yersiniabactin is blocked. Yersiniabactin production was tested using a GFP reporter assay with the knockout strain WA-CS *irp1*::Kan^r^ containing the pCJG3.3N plasmid as the reporter strain to detect yersiniabactin in supernatants. The number of bacteria versus the measured arbitrary amount of fluorescence from 50,000 counted bacteria is shown in panel A. A) Supernatants from the *irp2* knockout (E4Δ*irp2*) cultures (grey) induced a lower amount of GFP compared to supernatants from the wild-type (WT) cultures (white). This indicates that production of yersiniabactin was blocked in the *irp2* knockout. The experiments were performed in duplicate. B) The GFP reporter assay is dependent on yersiniabactin production, and production of yersiniabactin in the *irp2* knockout is blocked. Yersiniabactin production is only seen in the wild-type (WT) cells cultured in iron-depleted medium (NBD), while the *irp2* knockout (E4Δ*irp2*) strain cultured in NBD and iron-containing medium (NB), as well as the wild-type strain grown in NB, did not produce yersiniabactin. Three different experiments were performed, with each sample analyzed in duplicate. C) The expression of HMWP1 and HMWP2 is disrupted by the insertion of a kanamycin resistance gene into *irp2* using the Tagetron Knockout System. The *irp2* knockout (E4Δ*irp2*) strain was not able to produce HMWP1 and HMWP2 when cultured in NBD. M: marker; lane 1: wild-type strain E4; lane 2–5: *irp2* gene knockouts created using the wild-type strain E4; lane 6 wild-type EHOS strain 03-702; lane 7: wild-type EHOS strain 03-819 served as HPI-positive control because the HMWP2 of this strain was confirmed by Edman degradation.

Based on the observed increase in the virulence of HPI-positive isolates, it has been suggested that in addition to providing iron to bacteria, the HPI also contributes to the increased virulence of HPI-containing *Enterobacteriaceae*. To study the possible role of yersiniabactin in virulence, a knockout EHOS strain was successfully constructed. The disruption of the *irp2* gene was verified by DNA sequencing (Accession number: FN421462). A GFP production assay in which GFP is induced in the presence of yersiniabactin was used to confirm that the production of yersiniabactin was disabled in the *irp2* knockout. The fluorescence signal with supernatant from cultures of the *irp2* knock-out strain was low compared to the fluorescence signal obtained with the supernatant from wild type E4 cultures (Figure 1A). This indicates that yersiniabactin is produced by the wild-type E4 but not by the *irp2* knock-out isolate. When the supernatant from wild-type bacterial cultures grown in iron-containing medium was used for the GFP assay, the production of GFP was not upregulated, demonstrating that yersiniabactin production is dependent on iron (Figure 1B). In addition, no production of HMWP1 or HMWP2, as measured using SPS-PAGE, could be detected in the *irp2* knockout strain cultured in iron-depleted medium (Figure 1C), while HMWP1 and HMWP2 were expressed in the wild-type EHOS cultures. The putative HMWP2 band from EHOS 03-819 was confirmed by Edman degradation sequencing of the first eight amino acids, which were enough to identify HMWP2.

**Figure 2:**
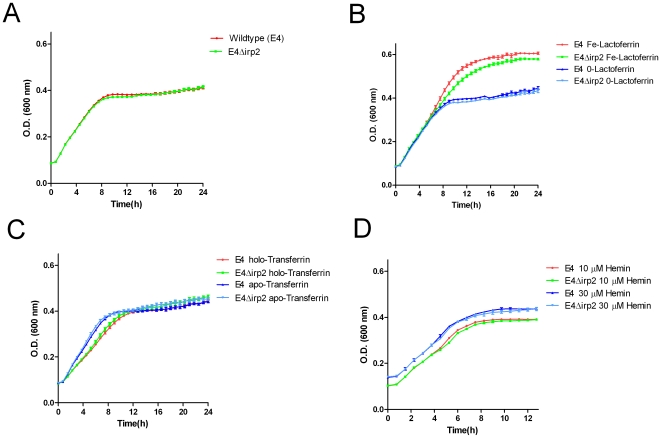
Growth curves of the EHOS E4 isolate and its corresponding *irp2* knockout strain. The results are presented as the mean of three experiments performed in duplicate on different days. Cells were grown in: A) nutrient broth treated with 1% Chelex; B) nutrient broth treated with 1% Chelex and supplemented with 80% iron-saturated lactoferrin (∼6.2 µM, Fe-LF) or unsaturated lactoferrin (∼6.4 µM,0-LF); C) nutrient broth treated with 1% Chelex and supplemented with 17 µM holo-transferrin or 17 µM apo-transferrin; D) nutrient broth treated with 1% Chelex and supplemented with 10 µM or 30 µM hemin.

The growth of the wild-type E4 and *irp2* knockout strains in the presence of different iron sources was tested. At low iron concentrations (nutrient broth treated with 1% Chelex), isolate EHOS E4 demonstrated slightly better growth during the stationary phase than the *irp2* knockout strain (Figure 2A). Furthermore, the time the wild-type strain remained in the exponential growth phase was longer, and the plateau of the stationary phase was significantly higher (*p*<0.001) for isolate E4 than the *irp2*-gene knockout in NB treated with 1% Chelex containing 0.54 mg/mL 80% iron-saturated lactoferrin as the main iron source (Figure 2B). This effect was abolished when iron-saturated lactoferrin was replaced with unsaturated lactoferrin. These results indicate that a strain with a functional HPI can obtain iron from saturated lactoferrin in the absence of free iron. No advantage for the E4 isolate compared to the knockout strain was observed in media containing either hemin or holo-transferrin as the main iron source (Figure 2C–D).

### Yersiniabactin Inhibits the ROS Response of Human PMNs

**Figure 3:**
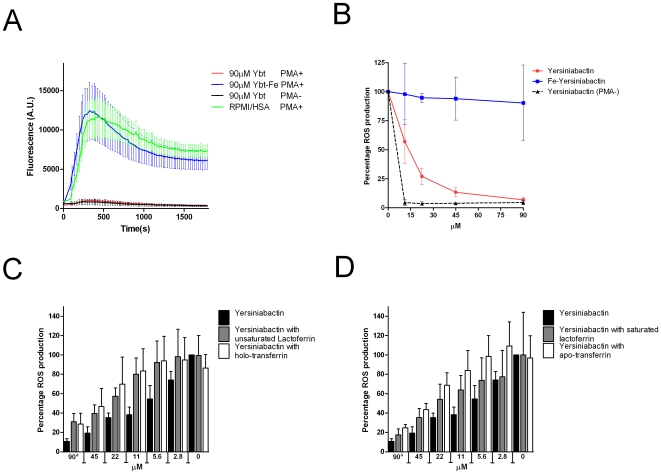
ROS production by PMNs is reduced by yersiniabactin. To determine the effect of yersiniabactin on the ROS production of PMNs, PMNs were pre-incubated with yersiniabactin. PMNs were then activated by PMA, and the production of ROS was measured using luminol. A) Absolute ROS production measured in arbitrary fluorescence units (A.U.) of PMNs after pre-incubation with yersiniabactin or iron-saturated yersiniabactin or the controls (PMNs that were only pre-incubated in RPMI/HSA medium and PMNs that were pre-incubated with yersiniabactin but not stimulated with PMA). B) The yersiniabactin-mediated inhibition of ROS production by PMNs is concentration dependent. Red line: the concentration-dependent decrease in production of ROS following treatment with yersiniabactin. Blue line: no decrease in ROS production following treatment with different concentrations of iron-saturated yersiniabactin. Black dotted line: negative control (PMNs incubated with yersiniabactin, but not stimulated with PMA); ***p value<0.0005, **p value<0.005, *p value<0.05. C) Treatment of PMNs with unsaturated lactoferrin or holo-transferrin partly inhibits the yersiniabactin-mediated inhibition of ROS production. Black bars: relative ROS production following treatment with yersiniabactin; gray bars: relative ROS production following treatment with yersiniabactin and unsaturated lactoferrin; white bars: relative ROS production following treatment with yersiniabactin and holo-transferrin. ^a^Concentration of the iron chelator. D) Treatment of PMNs with saturated lactoferrin or apo-transferrin partly inhibits the yersiniabactin-mediated inhibition of ROS production. Black bars: relative ROS production following treatment with yersiniabactin; gray bars: relative ROS production following treatment with yersiniabactin and saturated lactoferrin; white bars: relative ROS production following treatment with yersiniabactin and apo-transferrin. All experiments were replicated three times, and each sample was analyzed in duplicate.

As our hypothesis is that the ROS response of PMNs is dependent upon iron from lactoferrin, the effect of yersiniabactin on the ROS response was investigated. ROS production by PMNs following 4-β-phorobol-12-myristate-13-acetate (PMA) stimulation was inhibited in a concentration-dependent manner by unsaturated yersiniabactin, while iron-saturated yersiniabactin did not reduce ROS production by PMNs (Figure 3A, B).

The yersiniabactin-mediated inhibition of ROS production was reduced by the addition of unsaturated lactoferrin or holo-transferrin (Figure 3C). The results obtained with iron-saturated lactoferrin and apo-transferrin were comparable to those obtained with yersiniabactin (Figure 3D). These results demonstrate that the yersiniabactin-mediated repression of the ROS response of PMNs can be released by making iron available to the PMNs.

### Yersiniabactin Is Highly Effective Compared to Other Siderophores

**Figure 4:**
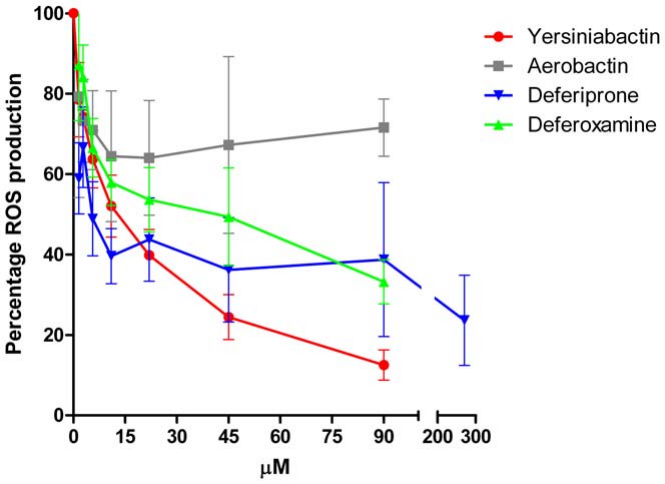
Other iron chelators also reduce ROS production by PMNs. Along with yersiniabactin, aerobactin, deferoxamine, and deferiprone also reduce ROS production by PMNs. To determine the effect of other iron-binding molecules on ROS production by PMNs, PMNs were pre-incubated with aerobactin, deferoxamine, deferiprone, or yersiniabactin. The pre-treated PMNs were then activated using PMA, and the production of ROS was measured using luminol. Red line: the concentration-dependent decrease in ROS production following yersiniabactin treatment. Gray line: the decrease in ROS production following treatment with aerobactin. Yellow line: the concentration-dependent decrease in ROS production following deferiprone treatment. Green line: the concentration-dependent decrease in ROS production following deferoxamine treatment. The level of ROS produced by stimulated PMNs without additives was set as 100%. All experiments were replicated three times, and each sample was analyzed in duplicate.

The effect of other siderophores on the reduction of the ROS response was examined. Yersiniabactin reduced ROS production more than aerobactin, which is also a known virulence factor in *Enterobacteriaceae* (Figure 4). Therapeutic iron chelators, such as deferoxamine and deferiprone, reduced the ROS production, but appeared to be less effective than yersiniabactin. Higher concentrations of deferiprone were also tested because deferiprone binds iron at a ratio of 1 iron to 3 deferiprone molecules, while the other tested iron chelators bind iron at 1∶1 ratio; however, the reduction of ROS production by 270 µM deferiprone was still less than the reduction following treatment with 90 µM yersiniabactin. When N-Formyl-L-methionyl-L-leucyl-L-phenylalanine (fMLP), rather than PMA, was used to stimulate the PMNs, similar results were observed (data not shown).

### Yersiniabactin Inhibits ROS Response of Human PBMCs and the Mouse Macrophage Cell Line J774.A1

**Figure 5:**
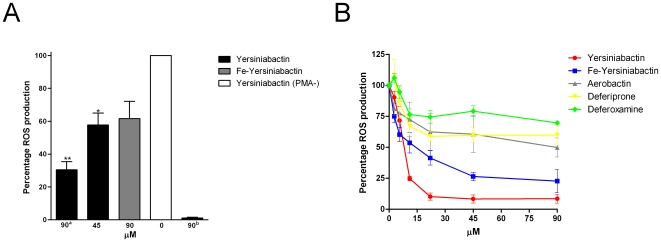
ROS production by PBMC-derived monocytes and in the J774A.1 cell line (mouse macrophages) is also reduced by pre-incubation with iron-binding molecules. A) ROS production in PBMC-derived monocytes pre-incubated with yersiniabactin or iron-saturated yersiniabactin. Black bars: PBMCs pre-incubated with 90 µM yersiniabactin and subsequently activated with PMA. Gray bar: PBMCs pre-incubated with 90 µM iron-saturated yersiniabactin and subsequently activated with PMA. White bar: no compounds added. ^a^Bar: Negative control: 90 µM yersiniabactin was added but the cells were not stimulated with PMA; **p value = 0.0053, *p value = 0.02. B) Yersiniabactin, aerobactin, deferoxamine, and deferiprone all inhibit ROS production in the J774A.1cell line (mouse macrophages) in a concentration-dependent manner, but yersiniabactin is the most potent in reducing ROS production. Red line: concentration-dependent decrease in ROS production following treatment with yersiniabactin. Blue line: concentration-dependent decrease in ROS production following treatment with iron-saturated yersiniabactin. Gray line: concentration-dependent decrease in ROS production following treatment with aerobactin. Yellow line: concentration-dependent decrease in ROS production following treatment with deferiprone. Green line: concentration-dependent decrease in ROS production following treatment with deferoxamine. The level produced by stimulated PMNs without additives was set as 100%. All experiments were replicated three times, and each sample was analyzed in duplicate.

Yersiniabactin significantly reduced the ROS production of PMA-stimulated monocytes (Figure 5A), while the reduction of the ROS production by iron-saturated yersiniabactin was not significant. Ten to twenty percent of the isolated peripheral blood mononuclear cells (PBMCs) were monocytes, which indicates that for each experiment the number of monocytes varied from 1×10^5^ to 2×10^5^.

No measurable ROS were produced by the human macrophage-like cell lines U937 and THP-1 [36], [37] following activation with PMA. In contrast, the mouse macrophage cell line, J774.A1, produced ROS following stimulation with PMA[38]. Furthermore, the production of ROS by PMA-stimulated J774.A1 cells was reduced by all iron chelators tested: yersiniabactin, aerobactin, deferoxamine, and deferiprone; however, as before, the ROS production was most strongly inhibited by yersiniabactin (Figure 5B). Importantly, no cytotoxicity was observed for any of the tested iron chelators (90 µM) against PMNs, PBMCs, or J774A.1 cells (data not shown).

## Discussion

The yersiniabactin iron uptake system encoded by the HPI is a known virulence factor of *Y. pestis* and other *Enterobacteriaceae*. The siderophore produced by *Enterobacteriaceae*, yersiniabactin, is a highly effective iron chelator that provides iron to the bacteria during infection. In this study we demonstrate that the growth of a yersiniabactin-producing strain of *E. hormaechei* was enhanced compared to a knockout strain in which the yersiniabactin production was disrupted in medium where iron-saturated lactoferrin is the main iron source. In media with hemin or holo-transferrin as the main iron source, yersiniabactin provided no additional benefit, probably because the tested strain, *E. hormaechei*, contains additional iron uptake systems.

Because it has been suggested that iron is important in the production of ROS, we investigated if yersiniabactin and other iron chelators influenced the production of ROS.

Yersiniabactin and other iron chelators reduced ROS production in PMNs, monocytes, and a mouse macrophage cell-line (J774A.1), which suggests that the decreased amount of iron available for the PMNs was responsible for the reduced ROS production. This is likely due to competitive binding for iron between yersiniabactin and iron-binding proteins produced by mammals. However, as yersiniabactin is a more effective iron chelator than lactoferrin or transferrin, the equilibrium is in favor of yersiniabactin, thereby blocking the iron supply to cells of the innate immune system. The connection between reduced iron uptake and reduced production of ROS by PMNs has been previously shown for deferoxamine and deferiprone [35], [39]. However, this also suggests that iron is necessary for the majority of ROS production by PMNs, monocytes, and macrophages. This also suggests that the Haber-Weiss reaction occurs and that hydroxyl radicals are formed after activation of these innate immune cells. Clearly, innate immune cells (PMNs, monocytes, and macrophages) produce endogenous hydroxyl radicals.

Myeloperoxidase catalyzes the conversion of hydrogen peroxide to hypochlorous acid (HOCl), which is thought to be the primary compound responsible for oxidative killing [30]. Surprisingly, however, myeloperoxidase deficiency does not lead to susceptibility to bacterial infections [30]; the endogenous production of hydroxyl radicals by innate immune cells could potentially explain this observation.

Several siderophores were tested: yersiniabactin, aerobactin, deferoxamine, as well as the iron-chelator deferiprone. Among these, yersiniabactin was the most effective in reducing ROS production by human PMNs, human monocytes, and the mouse macrophage-like cell-line J774.1A. Clearly, yersiniabactin is highly effective in reducing the innate immune responses of the host. Previous studies have shown that the presence of HPI increased the virulence of *E. coli* and *Yersinia* species in mice [4], [12], [13], [40]. In both a mouse bubonic model and a primary pneumonic mouse model, bacterial genes involved in the detoxification of ROS were down-regulated, indicating no ROS response targeted the bacteria. In contrast, the expression of yersiniabactin-iron uptake-system genes were strongly up-regulated [41], [42]. These results, combined with the results presented in this study, suggest that yersiniabactin reduces ROS production, most likely hydroxyl radical production in particular, in innate immune cells both *in vitro* and *in vivo*.

Clearly, ROS production, an effective killing mechanism of cells of the innate immune system [30], is inhibited by yersiniabactin. The effect of aerobactin, another siderophore produced by *Enterobacteriaceae*, is limited in its capability to reduce ROS production by PMN, monocytes, and mouse macrophages. This difference can be partly explained by the fact that yersiniabactin has a much higher affinity for iron than aerobactin (yersiniabactin, ∼4×10^36^ M^−1^ and aerobactin,1×10^23^ M^−1^)[43], [44].

Furthermore, we demonstrated that yersiniabactin is more effective than known and therapeutically used iron chelators, such as deferoxamine and deferiprone, in reducing ROS production by PMNs, monocytes, and mouse macrophages. Similar to aerobactin, both deferoxamine and deferiprone have a lower affinity for iron than yersiniabactin (deferoxamine, ∼1×10^30.6^ M^−1^ and deferiprone, ∼1×10^19^ M^−1^ [45], [46]). However, in addition to the iron-binding capacity, other factors (e.g., lipophilicity, pH stability, and solubility) may play a role. For example, enterobactin has a very high iron affinity (1.10^52^ M^−1^); however, its role in iron binding during infections is questionable because its solubility is very limited in aquatic solutions [43]. Furthermore, siderophores can be inhibited by lipocalin 2, which is a component of the innate immune system that limits bacterial growth by sequestering siderophores [47]. However, based on the resemblance of the yersiniabactin structure to the siderophore pyochelin, it is unlikely that yersiniabactin is bound by lipocalin 2 [34]. Clearly, the results presented here demonstrate that yersiniabactin is the most effective of the investigated siderophores in reducing ROS production under physiological conditions.

This suggests that yersiniabactin, or derivates of it, may be good candidates not only for the treatment of iron overload diseases, such as beta-thalassemia major, but also for reducing oxidative stress in chronic immune-related diseases, such as asthma or chronic obstructive pulmonary disease (COPD) [48]. In addition, many degenerative diseases have been correlated with altered ROS production [49]. Therefore, yersiniabactin may be also useful in reducing the progression of these degenerative diseases, such as Alzheimer's disease and spinal muscular atrophy [49], [50]. Furthermore, vascular diseases and type 2 diabetes have also been shown to have a relationship with ROS production, and yersiniabactin may also be able to attenuate the development and progression of these diseases [51]. However, a possible drawback for therapeutic use of yersiniabactin is that it may induce infections with bacteria that are able to take up iron-saturated yersiniabactin, as has already been shown for deferoxamine [35], [52], [53]. Additionally, as deferoxamine has also been shown to have immunosuppressive effects on T cells [54], which is mainly mediated through iron sequestering, yersiniabactin may also have immunosuppressive qualities.

In conclusion, even compared to lactoferrin, the mammalian protein that binds iron most strongly, the equilibrium for iron binding is in favor of yersiniabactin. Therefore, yersiniabactin not only provides bacteria with iron during infection, but also reduces the iron supply to cells of the innate immune system. Therefore, little or no iron is available for cells of the innate immune system, which need iron to produce hydroxyl radicals via the Haber-Weiss reaction. While it is controversial if the Haber-Weiss reaction takes place under physiological conditions, the fact that all tested iron chelators reduce, at least to some extent, ROS production in activated innate immune cells supports the hypothesis that this reaction can take place under physiological conditions. The reduced production of hydroxyl radicals would result in the decreased killing of bacteria by innate immune cells, thereby increasing the likelihood that yersiniabactin-producing *Enterobacteriaceae*, like the EHOS, would cause systemic infections. Therefore, we hypothesize that the increased virulence of HPI-positive *Enterobacteriaceae* is caused by yersiniabactin, which in addition to improving bacterial growth, also hampers ROS production in activated innate immune cells. The increased pathogenicity of the high-pathogenicity group of *Yersinia* compared to the low-pathogenicity group and the non-pathogenic group can be explained by the fact that yersiniabactin, which is only produced by the high-pathogenicity group, not only provides iron to the bacterium that is normally bound to iron-binding proteins produced by mammals, like lactoferrin, but more importantly, prevents ROS production by innate immune cells

## Materials and Methods

### Construction of the *irp2* Knockout Strain

To determine the role of the HPI in iron uptake, an *irp2* knockout strain was generated from the pQC-negative *Enterobacter hormaechei* outbreak strain E4 using the TargeTron knockout system (Sigma-Aldrich, St. Louis, MO, USA). This system inserts a kanamycin gene into the gene of interest, thereby disrupting its function. The manufacturer's protocol was followed, with some adaptations. In short, a *bla*_MIR-1_ promoter was introduced in front of the intron RNA to replace the T7 promoter using PCR. This modified pACD4K-C vector was transformed by heat-shock into competent *E. coli* DH5ß. The transferred vector was checked by DNA sequencing. The constructed plasmid was then transformed by heat-shock into a competent EHOS E4. After culture and selection of possible transformants, the isolates were checked for the correct insertion of the kanamycin resistance gene into the *irp2* gene by DNA sequencing. A GFP assay and SDS-PAGE were performed to determine whether production of yersiniabactin and HMWP1 and HMWP2, respectively, were blocked.

### HMWP1 and HMWP 2 Expression

To test the functionality of the HPI iron uptake system, the expression of HMWP1 and HMWP2 were monitored under various conditions using SDS-PAGE. Bacteria were grown in M9 minimal medium containing 60 mM Na_2_HPO_4_, 22 mM KH_2_PO_4_, 8.6 mM NaCl, 2 mM MgSO_4_, 0.1 mM CaCl_2_, 2 g/L glucose, and 5 g/L casamino acids (pH 7.4) (M9) and in iron-depleted M9. Iron was depleted with 1% Chelex-100 for 48 h at 37°C followed by the addition of 0.5 mM α,α′-dipyridyl (Sigma-Aldrich). Proteins were extracted using sonication followed by centrifugation at 50,000×*g* for 1 h at 4°C. The pellet was incubated for 1 h in 2 mL 1% SDS and then centrifuged at 50,000×*g* for 1 h at 4°C [55]. Subsequently, the supernatant was concentrated using a Centricon RC-YM 100 2-mL centrifugal filter (Millipore, Billerica, MA, US). Proteins were separated using 7.5% SDS-PAGE at 40 mA for 1 h, as described by Laemmli et al. [56]. The gel was stained with Coomassie Blue R. To confirm the identity of HMWP2, the proteins in the gel were electro-blotted onto a Immobilon-p Transfer Membrane (Millipore). The membrane was stained with Coomassie Blue R, and the suspected HMWP2 band was cut out and sequenced using Edman degradation (the Sequence Center, Utrecht, The Netherlands).

### Yersiniabactin Green Fluorescent Protein (GFP) Reporter Assay

To test the expression of the iron uptake system, yersiniabactin production was tested using a GFP-reporter assay [2], [40], [57]. The presence of yersiniabactin in the medium can be determined by measuring the production of GFP because 267 amino acids of FyuA were fused with GFP, and FyuA is upregulated in the presence of yersiniabactin. The knockout strain *Yersinia enterocolitica* WA-CS *irp1*::Kan^r^, which contains the pCJG3.3N plasmid, (kindly provided by Prof. J. Heesemann and Dr. S. Schubert) was used as the reporter strain to detect yersiniabactin in supernatants.

Tested isolates were cultured for seven days at 37°C under continuous shaking at 150 rpm in either 10 ml Nutrient Broth (NB), Difco nutrient broth (Becton Dickinson, Sparks, MD, USA) with 85.6 mM NaCl or 10 ml NB with 200 µM α,α′-dipyridyl (NBD). The supernatant was filtered, and 450 µl was added to 50 µl of a culture of the reporter strain WA-CS *irp1*::Kan^r^ containing the pCJG3.3N plasmid. This culture was grown at 28°C overnight in 10 ml NB with 200 µM α,α′-dipyridyl (NBD). The culture was then centrifuged, and the cells were resuspended to an optical density of 0.1 at 660 nm. When the tested supernatant contains yersiniabactin, GFP production will be induced the reporter strain that encodes the *fyuA*-*gfp* fusion construct, which is induced by yersiniabactin. The cultures with the GFP strain were incubated at 28°C overnight, washed, and diluted in phosphate buffered saline (PBS). The bacteria-associated fluorescence and scatter data for 50,000 gated bacteria were measured using flow cytometry (FACSCalibur, Becton Dickinson, Franklin Lanes, NJ, USA), and the mean fluorescence was determined.

### Growth During Iron Depletion

To test the hypothesis that competitive binding for iron occurs between yersiniabactin and other iron binding (or containing) proteins in low iron environments, the growth of the EHOS strain (E4) and its *irp2* gene knockout in different media was compared. NB was depleted of iron using a 1% Chelex treatment for 2 h (NBC). To obtain growth curves approximately 10^7^ cfu/mL EHOS E4 and the *irp2*-knockout were cultured in NBC supplemented with 0.54 mg/mL (∼6.2 µM) 80% saturated lactoferrin, 0.50 mg/mL (∼6.4 µM) unsaturated lactoferrin, 17 µM apo-transferrin, 17 µM holo-transferrin, 10 µM hemin, or 30 µM hemin (all Sigma-Aldrich) at 37°C under continuous shaking. The optical density at 600 nm was automatically measured every 20 min using a Bioscreen C MBR, an automated microbiology growth analysis system, according to the manufacturer's guidelines (Oy Growth Curves Ab, Helsinki, Finland). The data for each growth curve were subsequently natural log transformed. The slope of the exponential growth phase and the mean value of the stationary phase were determined using GraphPad Prism version 5.00 (GraphPad Software, San Diego, CA, U.S.). A paired *t*-test was then performed to compare the slopes and the mean values of the stationary phases from the different cultures [58].

### Isolation of Polymorphonuclear Leucocytes and Blood Mononuclear Cells (PBMCs)

Human PMNs and PBMCs used were isolated using a Ficoll-Histopaque gradient [59]. The percentage of monocytes in the PBMC fraction was determined using flow cytometry (FACSCalibur).

### ROS Production

To determine whether the ROS response of innate immune cells is affected by the presence of yersiniabactin or other iron-binding compounds, a chemiluminescence assay was used. First, 1×10^5^ PMNs were pre-incubated with the compound(s) of interest for 20 min at 37°C. The compounds used were: yersiniabactin, iron-saturated yersiniabactin, aerobactin (all Genaxxon BioScience GmbH, Biberach, Germany), deferoxamine mesylate (Novartis, Arnhem, The Netherlands), deferiprone (Duchefa Biochemie, Haarlem, The Netherlands), and iron-saturated and unsaturated lactoferrin and apo- and holo-transferrin. The reaction was placed in an LB 960 Microplate Luminometer Centro (Berthold Technologies, Bad Wildbad, Germany). Automatically, 0.2 µM 5-amino-2,3-dihydro-1,4-phthalazinedione (Luminol, Sigma-Aldrich) was added (final concentration 100 nM). After the first read, the PMNs were activated with PMA (Sigma-Aldrich). Following activation, the luminescence was measured for 0.3 sec every 50 sec over the course of 1 h. The area under the curve (AUC) was calculated using GraphPad Prism version 5.00. The results of each experiment were standardized by setting the AUC of PMNs without additives, but stimulated with PMA, as 100% ROS production.

To determine whether fresh human monocytes or mouse macrophages were able to produce hydroxyl radicals via the Haber-Weiss reaction and if yersiniabactin was able to reduce this response, the chemiluminescence assay was also performed with human PBMCs, human macrophage-like cell lines (U937 and THP-1), and a mouse macrophages cell line (J774A.1; ATCC TIB67) with some modifications [36]–[38]: the pre-incubation time was extended to 2 h, and the measurements were performed every 2 min for 2 sec over the course of 1 h for the human PBMCs and over the course of 1.5 h for the tested cell lines. All experiments were performed in duplicate and repeated three times.
